# Human Physiology

**Published:** 1841-04

**Authors:** 


### Art. XIII.

*Human Physiology.* By John Elliotson, m. d., f. r. s., &c. &c. *With which is incorporated much of the elementary part of the Institutiones Physiologicæ of J. F. Blumenbach*, m.d., f.r.s., *Professor in the University of Göttingen. Illustrated with numerous Woodcuts.* —*London*, 1835-40. 8vo pp. 1194.

The large space which we have been of late devoting to the discussion of physiological topics must be our excuse for not entering upon a full review of this work. The peculiarities of its character are such as to forbid our treating it with brevity if we enter at all into detail, and we are therefore compelled to dismiss it with such a general notice as may serve to convey to our readers an idea of its nature, and of the place which it holds in our estimation. We shall begin with a sketch of its history, which is not a little curious. In 1815, Dr. Elliotson translated the third edition of Blumenbach's "Institutiones Physiologicæ," anonymously, with the addition of twenty pages of notes; and in 1817, he published a new edition with his name and the addition of 150 pages of notes; subsequent editions were published in 1820 and 1824, in the last of which, the amount of Dr. Elliotson's notes greatly exceeded that of the text. Finding in the present edition his own matter would greatly exceed that of Blumenbach, and that much of the original would require emendation on account of recent discoveries, or might be better omitted, and that the disjointed nature of the work would be a source of greater inconvenience to the reader than ever, Dr. Elliotson resolved to remodel the whole, omitting many parts of the original, and blending his notes with as much of it as he could retain; and, as the portions of the original retained were of so much smaller amount than his own labours, and of a very elementary character, and as the proportions of Blumenbach's share and his own were thus completely reversed, Dr. Elliotson felt justified in giving his own name to the work. Now on this we must remark, that we do not think the formation of this kind of literary partnership, without the permission of one of the parties, at all justifiable. So long as Dr. Elliotson's additional matter remained in the form of notes, and Blumenbach's text was given unmutilated he had an undoubted right to append whatever he thought proper; but by putting his own name above that of Blumenbach on the title-page, and retaining just what suited his purpose of the original whilst his own new matter is not readily to be distinguished from it, he has in effect implicated that philosopher (in the mind, to say the least, of the indiscriminating reader) in the expression of his own opinions, with many of which we doubt not that the Professor of Göttingen would have been very sorry to have been identified.

Further, it is evidently impossible that a work thus concocted should have as much unity as one designed and executed by a single mind; and the bulky volume before us bears many indications of deficiency in that method and conciseness which are especially required in medical treatises, and of which Blumenbach's Institutiones were (at the time of their publication) an admirable specimen. Moreover, the long space, no less than five years, indeed more nearly six, which has intervened between the publication of the first and last parts, is fatal to the excellence of a work which, as its learned author doubtless hoped and expected, might, from its size and comprehensiveness, have been a standard treatise on the subject. The evil that has resulted from it is evident from the fact that, when the "small remaining part," which was for some years advertised as "just ready," at length made its appearance, it was found to contain nearly as much as the other two put together. Now this augmentation results (in part at least) from the very laudable desire on the part of the author, to give the fullest and most recent information on the subject to which it is devoted; but have not those discussed in the former parts advanced with equal rapidity, and is not the unity of the whole work thus completely destroyed? It is true that an Appendix might in some degree have rectified the evil, and an Appendix we find; but what does it contain? With the exception of a short account of Dr. Beaumont's experiments on digestion, it is entirely occupied with discussions on the author's two favorite subjects, Phrenology and Mesmerism, to which nearly fifty pages are here devoted, in addition to about eighty in the body of the work; and what is said of the former of these subjects almost exclusively concerns the relative personal merits of Gall and Spurzheim, instead of communicating to the enquiring student the progress of this department of science, and the evidence upon which its conclusions are based.

That the volume contains a vast amount of sound physiological information, and that it exhibits great learning and research, we most readily admit; but we cannot think it on that account a useful guide to the physiological student; for, whilst some of the subjects are fully and ably discussed, others of no less importance are very superficially handled, and the whole exhibits a want of that calm philosophical tone, for the absence of which no brilliancy or acuteness can compensate. The work is, nevertheless, a very amusing one, and is rendered so by the great variety of illustration which the author's various learning has enabled him to supply, (some of which, however, we are obliged to say, is of such a coarse description as a scientific work ought by no means to contain,) as well as by the very laughable egotism which pervades the whole, and which causes the learned doctor to launch out occasionally into tirades of a very ludicrous description, against all who differ from him. There is, for instance, a note of nearly four pages long, under the head of Phrenology, containing the history of all the improvements in medical practice to which Dr. Elliotson has contributed, and of the opposition which they at first encountered among the profession; the *gist* of which is to show that, as phrenology is laughed at by the ignorant and conceited, it is all the more true. A summary of this argument afterwards occurs under the head of Mesmerism; and we cannot give a better specimen of these amusing digressions than by quoting it.

"I have never yet declared an opinion upon a new truth that I have been obliged to retract. Phrenology has now advanced to its firm establishment; human glanders is universally admitted; auscultation is invariably practised except by the wretchedly ignorant; quinine, prussic acid, and creosote, are now in daily use. I stood abundant ridicule for advocating these, and will now stand more ridicule with the same firmness and the same silent pity or contempt which I have always felt for my opponents, till I see, as I shall, the truth of Mesmerism also admitted, and the world forget that it was ever doubted." (p. 686.)

The following piece of amusing sarcasm will serve as a specimen of the clever illustrations, not so remote from the subject or destitute of fundamental truth, with which the work is enlivened :

"The elderly man no longer likes new lights in science, nor improvements in institutions and methods. If he is a physician, he scorns new-fangled remedies and revolutionary discoveries in physiology and pathology, and tells of the number of follies he has seen prevail and pass away in his time; he acquires more confidence daily in nature's power of curing disease, being left daily more below the point of present knowledge, practising more and more feebly and uselessly, but more greedy than ever of fees, as though he was more informed and did more for his patients than ever. His feelings grow blunt, except his appetite for food and money; remembering his former pleasures, and being unsusceptible now of what he once was, he praises the past only, and condemns the present state of things, '*laudator temporis acti.*' " (p. 1022.)

Dr. Elliotson's work abounds in examples of similar acuteness in the mind of its author in regard to the faults of others, but he appears lamentably blind to his own. He reminds us much of the American original spoken of by Miss Martineau, (to whom, it is elsewhere related, this gentleman was thus purposely presenting a reflection of her own mind,) who told her that a long course of experience had convinced him that he was always in the right, but unfortunately he could not persuade other people to be of the same opinion. At the conclusion of the last part, he calls upon us to yield our assent to a case of transference of sensation, (a phenomenon which confessedly involves so many sources of fallacy, that no philosophic mind would credit it, except upon evidence of the most satisfactory nature,) upon the second-hand authority of his friend and assistant in Mesmeric experiments, Mr. Wood. This gentleman examined in Paris a case of clairvoyance of which the report had found its way to London, and having ascertained it to be a deception, he seems to be considered by Dr. Elliotson as proof against all further imposture, and as entitled to sway the belief of the whole scientific world. Mr. Wood then proceeded to Brussels to examine a case quoted by Mr. Townsend, and not being himself able to detect any fallacy, he came to the conclusion that the supposed phenomenon has a real existence. Dr. Elliotson endeavours to impart additional probability to the inference by a piece of reasoning, which will suffice to show his want of philosophic discrimination in questions of real intricacy. "There appears to me no reason why one part of the nervous system should not, in a new and strange state, acquire properties foreign to it, and possessed by another analogous part of the same system. The nutrient vessels of one organ will deposit in disease what is foreign to their natural function, and one secreting organ in preternatural states will secrete what nature never intended it should. I therefore know no reason why one nerve of sense should not be able to perform the function of another." (p. 1184.) Now we might admit this, and yet have scarcely advanced a step towards the belief in transference of sensation; for, it must be remembered that the formation of a sensory impression requires, not merely a particular conducting power in the nerve, but a particular apparatus for the production of a change in that nerve. Thus we can very well understand, how a nerve of common sensation might convey the impression of the presence of light, but Dr. Elliotson's explanation leaves us quite in the dark, as to how a definite image of an object can be formed, like that received by the retina, without an eye. Until, therefore, it is proved that this is unnecessary to vision, we must withhold our assent from the whole doctrine, even though Dr. Elliotson should treat *us* with the silent pity and contempt which he bestows so liberally on all who do not coincide with him.

We should not omit to say that, to the general reader, Dr. Elliotson's work will probably be very acceptable, partly through those very faults which unfit it for the professional student.

---

